# Nose-to-Brain (N2B) Delivery: An Alternative Route for the Delivery of Biologics in the Management and Treatment of Central Nervous System Disorders

**DOI:** 10.3390/pharmaceutics16010066

**Published:** 2023-12-31

**Authors:** Elizabeth J. Patharapankal, Adejumoke Lara Ajiboye, Claudia Mattern, Vivek Trivedi

**Affiliations:** 1 Medway School of Pharmacy, University of Kent, Central Avenue, Chatham Maritime, Canterbury ME4 4TB, UK; e.j.pathrapankal@kent.ac.uk (E.J.P.); l.ajiboye@kent.ac.uk (A.L.A.); 2 MetP Pharma AG, Schynweg 7, 6376 Emmetten, Switzerland; info@mattern-pharma.com

**Keywords:** CNS disorders, nasal delivery, biologics, nose-to-brain

## Abstract

In recent years, there have been a growing number of small and large molecules that could be used to treat diseases of the central nervous system (CNS). Nose-to-brain delivery can be a potential option for the direct transport of molecules from the nasal cavity to different brain areas. This review aims to provide a compilation of current approaches regarding drug delivery to the CNS via the nose, with a focus on biologics. The review also includes a discussion on the key benefits of nasal delivery as a promising alternative route for drug administration and the involved pathways or mechanisms. This article reviews how the application of various auxiliary agents, such as permeation enhancers, mucolytics, in situ gelling/mucoadhesive agents, enzyme inhibitors, and polymeric and lipid-based systems, can promote the delivery of large molecules in the CNS. The article also includes a discussion on the current state of intranasal formulation development and summarizes the biologics currently in clinical trials. It was noted that significant progress has been made in this field, and these are currently being applied to successfully transport large molecules to the CNS via the nose. However, a deep mechanistic understanding of this route, along with the intimate knowledge of various excipients and their interactions with the drug and nasal physiology, is still necessary to bring us one step closer to developing effective formulations for nasal–brain drug delivery.

## 1. Introduction

A growing number of central nervous system (CNS) disorders (e.g., caused by infection, injury, blood clots, age-related degeneration, cancer, autoimmune dysfunction, birth defects, multiple sclerosis, Alzheimer’s disease, Parkinson’s disease, meningitis, cerebral ischemia, etc.) are becoming more prevalent due to population growth and increased life expectancy. This poses a huge threat to patients and their families, as well as to society and the economy. These disorders require comprehensive treatment, which involves delivering therapeutics to the brain at appropriate levels to elicit a pharmacological response. However, despite the major advancements both in neuroscience and drug delivery research, the administration of drugs to the CNS remains challenging. In general, effectiveness-related issues arise when drugs cannot cross the blood–brain barrier (BBB). Therefore, currently, drugs with a low central bioavailability are applied by heavily invasive methods such as intrathecal and intracerebroventricular injection or by sensitive galenic approaches in oral dosage forms. Intranasal (IN) administration, on the other hand, serves as an alternative route for effective delivery to the CNS. It is non-invasive and can use nerve pathways for nose-to-brain drug transport to provide a fast onset of action, a possible reduction in systemic adverse effects, and higher bioavailability in the brain. Furthermore, the intranasal application is convenient for the patients, easier to apply in emergencies, and can save costs (e.g., reduced burden on trained medical and care staff).

Over the past decades, there has been significant progress in drug delivery and design by the pharmaceutical industry. However, areas focusing on the management of CNS disorders have considerably lagged [1]. The analysis conducted by Kesselheim, Hwang, and Franklin indicated a decline in CNS drug development since 1990, both in early and late-stage clinical trials [2]. Several factors, including an inadequate understanding of requirements for targeted CNS delivery, the complexity of both CNS physiology and diseases, increased drug development times and costs, and the higher risk of clinical failures, have severely limited the growth of new treatment possibilities for CNS disorders [1,3]. Moreover, the difficulty of poor drug transport across the BBB has been identified as the primary issue for the under-development of CNS pharmaceuticals [4,5,6]. It is widely accepted that most CNS disorders are unmanageable by non-invasive drug therapies because more than 98% of all potential CNS drugs do not cross the BBB. Therefore, researchers are now focusing on enhancing the delivery of potential therapeutics, including biomolecules, to the brain. This review provides a summary of challenges and specific approaches used to enhance both BBB permeability and drug bioavailability in the brain, with a specific interest in the use of large molecules (e.g., proteins, peptides, oligonucleotides, antibodies, steroids, and vaccines) via the possibility of direct nose-to-brain (N2B) drug delivery.

## 2. Potential of Biologics for the Management of CNS Disorders

The scope of therapeutic biologics to serve as an established first-line treatment of CNS disorders has rapidly evolved over the last few years because of their vast potential in managing these diseases. Table 1 highlights some of the biologics and their therapeutic applications in the treatment/management of CNS disorders.

## 3. Limitations Associated with Drug Delivery to the Brain

In contrast to other organs in the human body, the functioning of the CNS is distinctly defined by the presence of physiological barriers known as the BBB and blood–cerebrospinal fluid barrier (BCSFB) [31]. These physical, metabolic, and transporter-regulated barriers act to separate the CNS from the peripheral system by protecting it from any external toxins, stimuli, and foreign substances, including active pharmaceutical ingredients (APIs). Furthermore, the BBB maintains the homeostasis of the brain by selectively regulating the entry/exit of important nutrients, proteins, ions, and metabolites [32,33]. The limited permeability of the BBB is mainly attributed to its structure, which consists of brain capillary endothelial cells that are interconnected by tight junctions [34]. Typically, access to the brain via transcellular or paracellular mechanisms across the BBB is restricted to lipid-soluble small molecules with a molecular weight of <500 Da [4]. On the other hand, water-soluble substances with a larger size and positive charge could be transferred to the brain through alternative pathways such as receptor-mediated/adsorptive endocytosis or via transporter proteins. However, particularly for APIs, the active efflux transporters in the BBB, such as P-glycoprotein (P-gp), still pose a major obstacle to their delivery to the brain [35,36]. Also, the BBB comprises a metabolic barrier containing several enzymes (e.g., cytochrome p450) with the capacity to alter endogenous and exogenous molecules that could otherwise evade the physical barrier [37]. Therefore, a proper understanding of the physiological features of the BBB is important to be able to achieve effective brain transport of therapeutic agents.

As a result, several approaches have been attempted to either bypass or facilitate drug access across the BBB. These explorative strategies involve:

- BBB disruption that includes the temporary opening of tight junctions to enable passage through the BBB by optimizing the physio-chemical properties of therapeutic molecules [38,39,40,41,42,43,44,45].
- The use of drug delivery systems (DDS) and brain transport vectors for targeted BBB passage [46,47,48,49].
- Developing approaches to exploit various endogenous transport systems present at the BBB [50,51,52,53].
- Formulations to utilize alternative transport routes for direct brain delivery that can exclude the BBB [54,55,56].

Overall, it is important to develop novel approaches to enhance the delivery of APIs to the CNS and revolutionize the treatment of CNS disorders to improve patient outcomes. Amongst various approaches, intranasal delivery of drugs can be a promising approach to bypassing the BBB and delivering therapeutics directly to the brain.

## 4. Intranasal Drug Delivery

Several invasive techniques, including intraparenchymal, intraventricular, and intrathecal delivery, have been investigated to establish the direct transport of drug molecules to the brain [57]. However, these procedures may not be suitable for patients with chronic illnesses who require long-term treatment due to associated discomfort and the possibility of reduced effectiveness of the drug. The IN route of administration can provide a fast, pain-free, and non-invasive option for the delivery of drug substances to the CNS [58]. The large surface area of the nasal cavity and high vascularization of its mucosa can facilitate rapid drug absorption and fast onset [59]. Not to mention that the IN route also avoids harsh environmental conditions of the gastrointestinal (GI) tract and the first-pass metabolism. The possibility to exploit the nerve pathways after nasal administration also offers the unique opportunity of targeting drugs directly to the brain, making it highly attractive for the delivery of sensitive biotherapeutics [59].

**Figure 1:**
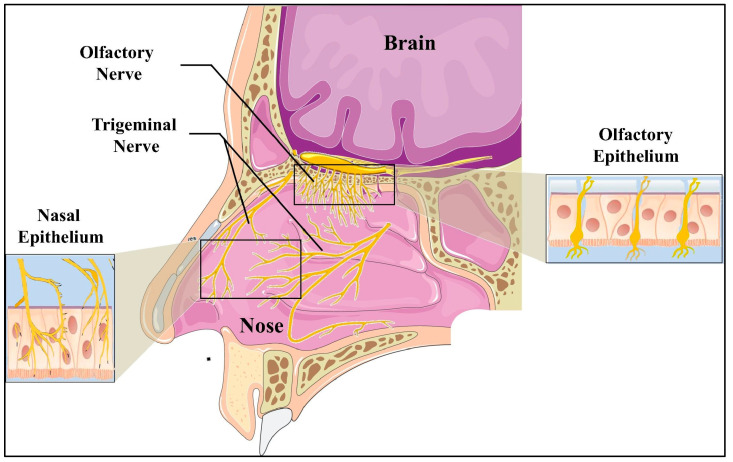
Illustration of the anatomy of the human nasal cavity (reprinted with permission from Elsevier) [60].

IN drug delivery is based on the unique physiology of the nasal cavity, which provides a direct connection between the external environment and the CNS. A simple illustration of the anatomy of the human nasal cavity is presented in Figure 1.

The details of nasal physiology have been comprehensively covered by various authors [61,62,63,64,65,66]. Nonetheless, features including the highly vascularized and permeable mucosal lining of the nasal cavity to allow for the rapid and efficient absorption of drugs and the availability of the olfactory and trigeminal nerve pathways are important to mention in this context. The olfactory area is directly connected to the brain (especially the olfactory bulb) via olfactory nerves. Along with this, the respiratory region is supplied with trigeminal sensory neurons and blood vessels [67]. Through a direct neuronal pathway, drugs may enter into different regions of the brain, providing a strategy to overcome the BBB. The exact mechanism of drug transport from the nasal cavity to the brain is still a topic of discussion, but some authors describe that the presence of transporters both in the olfactory bulb and respiratory mucosa of the nasal cavity may play an important role [68,69,70].

### 4.1. Challenges Associated with IN Delivery

There are various challenges associated with the IN delivery of drugs that include limited size of the nasal cavity, nasal mucus, mucociliary clearance (MCC), and enzymatic degradation but also changes in the nasal anatomy, e.g., polyps. The mean volumes of the nasal septum left/right nasal cavity, left/right inferior nasal conchae, and left/right middle nasal conchae are about 5 cm³, 7.6 cm³, 3.1 cm³ and 1.3 cm³, respectively, but gender and age differences can be statistically significant [71]. Therefore, nasal drug delivery is limited by the applicable volume of about 150 µL per nostril for adults and is potentially mainly suitable for high-potency drugs. If the instilled volumes exceed the limited capacity of the nose, the administered preparations are partially swallowed, or they simply run out of the nose.

Nasal mucus consists of a lower, liquid layer (“periciliary liquid”) that is covered by a more viscous gel phase and includes a thin layer of surfactant that spreads mucus all over the epithelial surface. Mucus contains inorganic salts, antimicrobial enzymes, immunoglobulins, and glycoproteins [72]. It is slightly acidic (pH 5.5–6.5), required for optimal ciliary clearance, and has limited buffering capacity [73]. The nasal mucus plays an important role in mediating immune responses to allergens and infectious particles by trapping them as they enter the respiratory passage [74,75,76,77,78,79,80,81]. MCC is the self-cleaning mechanism of the airways and a protective process for the lungs in removing inhaled particles, including pathogens. Within the thin periciliary liquid layer, the cilia (tiny hairs) beat in a coordinated fashion directed to the pharynx and create motions that drain mucus from the nasal passage to the throat, where it is swallowed and digested by stomach juices or removed by blowing the nose. Effective MCC depends on factors such as the number of cilia, their structure (particularly their height), and especially the quality of the mucus. On the other hand, particle transport by MCC may restrict the absorption of medication in aqueous formulations to an estimated 20–30 min. If the formulation irritates the nasal mucosa, this causes the irritant to be rapidly diluted, followed by increased clearance.

The in vivo clinical or pre-clinical (animal) experiments are particularly challenging when it comes to IN delivery. For example, the application of mild anesthetics is very common during IN studies that, in some instances, could result in different brain delivery and pharmacokinetics due to the activation of the glymphatic system [82,83]. Therefore, in vivo microdialysis experiments in freely moving animals could be considered in such cases [84]. Other options, such as laser scanning fluorescence microscopy, positron emission tomography (PET), and nuclear magnetic resonance spectroscopy (MRS), provide an elegant option for the evaluation of the distribution of nasally applied drugs with systemic/brain activity [85,86]. In contrast, traditional immunolabeling procedures require cutting the sample into thin sections, which restricts the ability to label and examine intact structures.

It is often difficult to estimate the results from publications since the exact galenical formulation is rarely apparent, information on the duration of the experiment (stability of the API over time, dosage regimen), and sampling of blood and tissue are limited [87]. Sometimes the test set-up also plays a role if unrealistically large volumes are applied or the IN formulation is not comparable to that of the oral/IP/SC applications.

The bioavailability of intranasally administered drugs can be greatly affected by enzymatic degradation, as the nasal mucosa contains a wide spectrum of xenobiotic-metabolizing enzymes [80,81]. Aldehyde dehydrogenases, cytochrome P450-dependent monooxygenases, rhodanese, glutathione transferases, epoxide hydrolases, flavin-containing monooxygenases, and carboxyl esterases have all been reported to occur in substantial amounts in the nasal cavity. These play a major role in the decomposition of actives in the nasal cavity. For example, oestradiol, testosterone, and decongestants are enzymatically degraded by cytochrome P450-dependent monooxygenases [26,80]. Although the impact of enzymatic degradation in the nasal mucosa remains inconclusive, it can be reduced to some extent either by enzyme inhibitors or by the saturation of enzymes.

### 4.2. Strategies to Enhance in Drug Delivery to the CNS

The selection of suitable excipients or formulations is critical for the effective IN delivery of actives, which becomes paramount for biologics. The hostile environment of nasal tissue, which is designed to protect the body from pathogens, makes delivery of complex biologics difficult. However, applications of agents such as permeation enhancers, mucoadhesive compounds, enzyme inhibitors, and vasoconstrictors can aid in increasing the efficacy of the IN formulations. As a general requirement, it is a must that an aqueous IN formulation is safe to deliver with respect to the nasal pH and osmolality. The pH of the healthy nasal epithelium is 5.5–6.5; a pH lower than 5.5 or higher than 6.5 may cause local adverse effects and affect drug permeation. The osmolality of the nasal solution should be 290–500 mOsm/kg; higher values are tolerable for emergencies or single applications, but isotonic formulations are important for chronic use, and hypotonic solutions should be avoided [88]. The section below briefly discusses existing strategies for promoting the absorption of therapeutics through the nasal cavity.

The selective IN permeation of small hydrophilic and lipophilic molecules is usually achievable, but the same cannot be said for high-molecular-weight actives as the nasal epithelia serves as a robust barrier for N2B transportation. This limitation can be addressed by increasing the nasal permeability with a permeation enhancer that can aid the transfer of biologics via neural or cellular pathways [89,90]. Permeation enhancers open up the tight junctions of the nasal epithelium either by swelling or by temporarily dissolving the membrane protein [64]. Furthermore, these are also known to improve drug solubilisation, reduce mucociliary clearance, limit enzymatic degradation, and increase the contact time of the drug with the nasal mucosa [91]. In general, they are classified according to their molecular weight, with linear or cyclic structures such as thiolated polymers. Low-molecular-weight compounds such as phospholipids, surfactants, bile salts, and their derivatives, as well as cyclodextrin, polymers (e.g., chitosan and carbopol), and cell-penetrating peptides (CPP), referred to as high-molecular-weight compounds, are commonly utilized as permeation enhancers [92,93,94].

The use of CPPs in particular has gained a lot of attention lately. The permeation enhancement mechanisms associated with CPPs are still debated in the literature, but electrostatic interactions between the positively charged CPPs and the plasma membrane are considered a possible first step to promote drug permeation [95,96]. The research by Ziegler et al. provided a complete overview of the cellular absorption efficiency of CPPs [97]. There is also evidence that CPPs containing unconventional stereochemical forms (D-from instead of L-form) can, on occasion, provide greater resistance to enzymatic degradation [98]. Therefore, CPPs in such cases can act as a permeability enhancer and also prevent the drug from enzymatic degradation. Low-molecular-weight permeation enhancers, on the other hand, are effective owing to their structural resemblance to the endothelial membrane. These agents can interfere with lipophilic as well as hydrophilic fractions due to their bipolar structures, thereby disrupting membrane bilayer integrity and promoting drug absorption [99].

Mucins are a prominent component of nasal mucus, and mucolytics (e.g., N-acetyl-cysteine) are needed to reduce the viscosity of the bronchial secretions and facilitate penetration of the drug by breaking disulphide crosslinks between mucin monomers [100,101,102]. With an average thickness of 10–15 μm, the nasal mucus layer is the upper respiratory tract’s first-line defensive barrier, hence maintaining a healthy airway and safeguarding the epithelium [103]. Thiol-containing fatty acids such as N-dodecyl-4-mercaptobutanimidamide and 2-mercapto-N-octylacetamide are reported to increase the mucus-penetrating capabilities of formulations such as self-emulsifying drug delivery systems (SEDDS) [103]. These formulations were shown to outperform equivalent SEDDS without thiols in terms of mucus permeation.

Mucoadhesive agents such as pectin, chitosan, and hydroxypropyl methylcellulose (HPMC) retain the therapeutic agent close to the site of absorption, resulting in a larger drug concentration gradient at the nasal epithelial membrane and hence increased absorption [104]. Depending on the functional groups present on the polymer backbone, mucoadhesives can improve absorption via enhanced nasal drug retention and/or decrease nasomucosal clearance [105]. Mucoadhesion primarily occurs through hydrogen bonding between the mucoadhesive polymer’s carboxylic acid groups and the hydroxyl groups that characterize mucus glycoproteins in the case of negatively charged polymers such as polyacrylic acid [106,107,108]. In addition, cationic polymers with a high density of positive charges (e.g., chitosan) can also interact with negatively charged mucus glycoproteins via electrostatic interactions, resulting in enhanced retention of the formulation at the delivery site. The use of vasoconstrictors (either in conjunction with a nasal formulation or as an excipient in the formulation) while targeting the olfactory region can also ensure increased drug concentration in the brain and limit systemic absorption [64].

Nasal mucosa includes a range of enzymes, including monooxygenase, reductase, transferase, and proteolytic enzymes, which can induce the degradation of drugs and limit their absorption. Incorporation of appropriate nanocarrier systems, such as polymeric nanoparticles or lipid-based nanocarriers (e.g., liposomes, solid-lipid nanoparticles (SLNs), nanostructured-lipid carriers (NLCs), nanoemulsions, lipid drug conjugates (LDCs), self-emulsifying drug delivery systems (SEDDS), etc.), is known to prevent the enzymatic degradation of drugs in the nasal cavity [109,110]. Other approaches, including PEGylation, have also been shown to protect biologics from degradation and can increase the half-life of a drug [111]. However, it should be noted that sometimes PEGylation might result in unexpected alterations in the biological activity of biologics. For example, the substrate selectivity of cholesterol oxidase was noted to change from total cholesterol to high-density lipoprotein (HDL) cholesterol following PEGylation. Similarly, the PEGylated growth hormone (pegvisomant) exhibited agonistic rather than antagonistic action compared to the non-PEGylated hormone [112]. So, in these cases, the protective effect of PEGylation was minimal; hence, this approach requires further investigation for the nasal administration of biologics.

Polymer-based drug carrier systems include polymeric nanoparticles, colloidal carrier systems, polymer–drug conjugates, and the application of a smart polymer-based system such as stimuli-sensitive hydrogels or in situ nasal gels, etc. [113,114]. In recent years, a number of biodegradable and biocompatible natural (e.g., alginate, chitosan) and synthetic (poly (lactic-co-glycolic acid) (PLGA), poly (acrylamide), poly (lactic acid) (PLA), poly (lysine), poly (caprolactone), and poly (acryl cyanoacrylate), etc.) polymers have been investigated to develop novel carrier systems for controlled and targeted CNS delivery via the nasal route [115,116,117,118].

Liposomes, nanoemulsions, lipid nanoparticles, SLNs, LDCs, and NLCs are also extensively utilized for nasal drug administration due to their biocompatibility and biodegradability [119]. Liposomes have been extensively investigated as carrier systems for therapeutic drug delivery to the brain. Salade et al. showed the use of chitosan-modified anionic liposomes for ghrelin nasal administration [120]. Similarly, the application of cationic liposomes instead of a typical solution for IN administration of a model protein (ovalbumin) showed increased bioavailability and activity in the brain at a substantially lower dosage [121]. Nanoemulsion can also be a promising system for N2B delivery because of its small droplet size, lipophilicity, biocompatibility, low toxicity, and greater permeability. The nanoemulsions containing zolmitriptan and quetiapine fumarate showed high brain targeting efficiency when delivered intranasally [122,123]. SLNs are considered more stable than liposomes, and because of their smaller size, they can be a viable option for N2B drug delivery [124]. For example, in one study, levofloxacin and doxycycline SLNs showed improved AUC and brain concentration compared to the simple nasal solution [124,125]. NLCs are second-generation SLNs that are characterized by higher drug encapsulation and improved stability. Chitosan-modified NLCs containing glial cell-derived neurotrophic factor (GDNF) showed improved therapeutic efficacy and resulted in considerable improvement in the 6-OHDA-lesioned rat model’s behavioral function, indicating a successful delivery of GDNF to the brain [126]. Efavirenz containing NLCs, when delivered intranasally as treatment for neuroAIDS, revealed a significant improvement in the drug distribution in the brain [127]. It is also worth mentioning that devices play a very important role in the IN delivery and targeting of APIs, but discussion on devices was considered out of scope for this review. Table 2 outlines the application of various approaches/excipients used in the IN delivery of APIs to target them to the brain.

## 5. In Delivery of Biologics to the CNS

A significant amount of work has been conducted on the suitability of the IN route for delivering high-molecular-weight therapeutics (e.g., peptides, proteins, nucleic acids etc) and various neurosteroids [171]. The susceptibility of biologics to enzymatic breakdown and their limited permeability through the epithelium via transcellular and paracellular pathways result in poor absorption of biologics from a mucosal site. As a result, they are often delivered through invasive and painful injections to boost their bioavailability. However, novel formulations and delivery techniques are being continuously developed to improve the administration of both small molecules and macromolecular therapeutics [172]. As previously noted, unlike parenteral administration, IN delivery is extremely easy and convenient for patients, making it particularly appealing for chronic treatments. The following sections discuss the formulation strategies used in the delivery of biologics in the treatment of CNS disorders.

The delivery of peptides to the brain has received growing interest in recent decades due to its pharmacological significance in the treatment of various CNS ailments, including neurodegenerative diseases, cancer, and ischemic strokes [173,174,175]. Insulin is one of the most extensively researched biologics in terms of its effects on the CNS after IN delivery. One of the earliest studies on peptide delivery to the brain involved the IN administration of an aqueous solution of insulin that showed pharmacological efficacy but also provided information on its limited transportation into the brain [176]. Since then, IN administration of insulin aqueous solutions has been extensively studied in various preclinical and clinical trials for the treatment of Alzheimer’s disease, mild cognitive impairment, diabetes, insulin resistance, and Parkinson’s disease, among other conditions.

The impact of excipients and formulation types is very important while developing an IN-drug delivery system. For example, Kamei et al. studied IN delivery of insulin solution using L-penetratin and D-penetratin (cell-penetrating, 16 mer peptide). They reported that the IN administration of radio-labeled insulin with L-penetratin in rats resulted in higher levels of insulin in the anterior region [177]. This finding was further confirmed in Alzheimer’s disease model mice, where co-administration of insulin with L-penetratin resulted in slower memory loss progression than co-administration of insulin with D-penetratin or with the administration of insulin alone [178]. In another study, Picone et al. developed negatively charged nanogels constructed of polyvinylpyrrolidone (PVP) that resulted in enhanced insulin delivery to the brain [179]. Maitani et al. investigated the permeability of insulin-entrapped liposomes through rabbit nasal mucosa and compared it with the permeability of insulin solution with/without pre-treatment with sodium glycocholate (GC) [180]. They reported a positive outcome of pre-treatment with GC, especially for insulin-containing liposomes (i.e., the liposomes penetrated more efficiently following pre-treatment with GC). Similarly, Morimoto et al. developed polyacrylic acid (PLA) gel using insulin and calcitonin for IN delivery to the brain [181]. They reported higher insulin absorption from 0.15 *w*/*v* PLA gel than that from 1% *w*/*v* gel after nasal delivery in rats, which could be related to the gel viscosity [181]. A similar study investigated the effects of putative bioadhesive polymer gels on nasal mucociliary clearance in rat models. The results showed that all formulations reduced IN mucociliary clearance, increasing the formulations’ resident duration in the nasal cavity [182].

Pringles et al. used dry insulin powder in deposition trials in rabbits to assess the effect of deposition patterns utilising different spray devices on insulin bioavailability [183]. The authors concluded that anterior deposition of the formulation in the nasal cavity results in maximum insulin bioavailability due to the high degree of surface coverage over the nasal epithelium. In another study, Nagai et al. investigated the absorption of dry powder insulin combined with microcrystalline cellulose (MCC) and other cellulosic derivatives, where MCC was shown to have the largest permeability-boosting impact [184]. It is understood that MCC could be able to bind with the calcium ions in the nasal epithelium to open up the tight junctions while temporarily hindering mucociliary clearance due to its mucoadhesive nature [185].

In a study conducted by During et al., where they dispersed [Ser(2)] exendin (1–9) [a glucagon-like peptide-1 (GLP-1R) receptor agonist] in a 10% β-cyclodextrin solution that is believed to act as a permeation enhancer, peptide solubilizer and stabilizer [186], an increase in learning and diminished kainic acid-induced apoptosis were observed in mice, most likely mediated by GLP-1R expression in the hippocampus. Similarly, Banks et al. investigated the brain distribution of the radioactively labeled GLP-1 antagonist exendin (9–39) (I-Ex) after IN and IV administrations [187]. An I-Ex solution in phosphate buffer or normal saline with or without cyclodextrin was utilized in this study. After IN administration, the results showed that olfactory bulb absorption of I-Ex was substantially faster than after IV administration, and it increased by roughly 60% when cyclodextrin was added. Kamei et al. prepared a formulation of exendin-4 with L-penetratin that resulted in the delivery of the peptide to the hypothalamus and hippocampus after the IN delivery [135]. These findings indicated that the IN exendin-4/CPP combined with the supplementary insulin resulted in a therapeutic response against severe cognitive deterioration in a senescence-accelerated animal model of cognitive dysfunction as tested via the Morris water maze test [135].

The IN delivery of proteins is equally gathering substantial interest amongst pharmaceutical scientists. For example, neurotrophic factors have enormous potential as protein therapeutics in the CNS, but their use has been severely limited due to delivery issues and systemic adverse effects. Insulin-like growth factor-1 (IGF-1) is one of the most effective proteins delivered to the brain via the IN route. Thorne et al. demonstrated that the IN administration of recombinant human IGF-1, wherein [^125^I]-IGF-1 was dispersed in PBS containing 0.25% BSA, resulted in substantially higher CNS concentrations of the drug than the equivalent IV dosage [188]. These studies were among the first to indicate widespread distribution of a protein inside the CNS, possibly by utilising the olfactory and trigeminal nerve pathways. Lin et al. [189] demonstrated that IN administration of recombinant human IGF-1 enhanced neurobehavioral functions, decreased apoptotic cell death, and boosted the proliferation of neuronal and oligodendroglial progenitors in neonatal rats 1 h after hypoxic-ischemic brain injury.

The 18 kDa polypeptide growth factor basic fibroblast growth factor (bFGF) exhibits neuroprotective effects in a variety of brain-related illnesses. In a study conducted by Zhang et al., bFGF coupled with functionalized Solanum tuberosum lectin NPs (STL–PEG–PLGA NPs) (120 nm and negative surface charge) was delivered intranasally in an AD mouse model. The results revealed that the IN administration of NPs increased the AUC of radio-labeled-bFGF by 1.5 times when compared to the free protein, and the modification with the targeting ligand enhanced the value of the AUC by up to 3 times more [150]. In an ischemic rat model, bFGF encapsulated in gelatine NLC (128 nm and negatively charged) comprising phospholipids, cholesterol, and Poloxamer 118, was evaluated. As compared to IV, the results showed 1.5 times more protein accumulation in different brain areas, as well as an improved therapeutic response [190]. The same nanocarrier was employed to deliver bFGF for PD treatment. The findings revealed high protein levels in various areas of the brain, including the olfactory bulb and striatum, as well as an improvement in their therapeutic effect after IN administration in a PD rat model, when compared to free protein and IV administration of the nanoencapsulated protein [156].

Monoclonal antibodies (mAbs) have received special attention among biologics recently, resulting in a rising number of therapeutic antibodies in clinical trials and even on the market [191,192]. As of 2021, the FDA had approved 103 therapeutic antibody drugs including the use of aducanumab (marketed as Aduhelm^®^) for the treatment of Alzheimer’s, clearing the path for future research into antibody-based treatments for other CNS diseases [193,194]. Nevertheless, only a few researchers have looked into IN delivery of an antibody to the CNS since their high molecular mass (~150 kDa) and polarity prevent BBB penetration. In a study, a TNF-α inhibitory single-chain Fv antibody fragment (scFv) (ESBA105) was delivered intranasally through Pz-peptide (4-phenylazobenzoxycarbonyl-Pro-Leu-Gly-Pro-DArg) in mice [195]. The addition of a penetration-enhancing peptide to the formulation increased the antibody distribution to the olfactory bulb and cerebrum while reducing systemic exposure. Similarly, anti-TRAIL antibodies adsorbed onto the surface of PLGA and NLC NPs were shown to swiftly and efficiently reach the CNS in mice following IN delivery. Another example includes the delivery of active-containing PLGA NPs coupled with mAb anti-EPH3 and trimethyl chitosan coating [196]. This strategy is based on the anti-EPH3 antibodies’ ability to target a membrane receptor that is overexpressed in the stroma and vasculature of gliomas. In a glioma rat model, the NPs were loaded with temozolomide and delivered intranasally. Although the antibody in this case is effectively used as an excipient, it still suggests that the nasal route can be suitable for the delivery of large molecules. Fluorescence imaging revealed that NPs functionalized with anti-EPH3 antibodies accumulated in the brain more than non-functionalized NPs. These results suggest that the IN route can be an excellent, simple, and effective non-invasive method in the treatment of CNS disorders such as AD [197].

The IN route has been similarly studied for the delivery of nucleotide-based drugs. The importance of oligonucleotide therapy in the treatment of chronic inflammatory respiratory diseases is comprehensively discussed in a review published by Mehta et al where they also emphasised the importance of delivery routes including nasal administration [198]. Many investigations have employed the olfactory pathway to transport oligonucleotides or oligonucleotide-loaded nanoparticles [199,200,201,202,203,204,205,206,207] to the brain. Current research has concentrated on cell-penetrating peptide (CPP)-based delivery methods for the treatment of neurodegenerative illnesses, which have significant transmembrane capabilities and tremendous therapeutic potential [208]. For example, CPP Tat linked to ethylene glycol-polycaprolactone copolymers (mPEG-PCLTat/siRNA nanomicelles) when delivered intranasally showed superior siRNA targeting to the brain while reducing systemic toxicity [199]. The findings suggested that mPEGPCL-Tat has a role in delivering greater levels of siRNA to the brain via a non-invasive IN route using the trigeminal and olfactory nerve pathways. The results also indicated that the findings might be used in the treatment of persistent neuropsychiatric illnesses, brain tumors, and cerebral infarction. Similarly, in a study by Yang et al., a cell-penetrating peptide (DP7-C) encapsulated with hyaluronic acid (HA) was developed to create multifunctional core-shell structure nanomicelles (HA/DP7-C) [200]. To test its efficacy in glioma, siRNA was encapsulated within the nanomicelles and delivered intranasally to rats. In the in vitro studies, the nanomicelles demonstrated high cell uptake and minimal cytotoxicity. In vivo investigations revealed that IN delivery of the HA/DP7-C siRNA reached the CNS via the trigeminal nerve route within hours. Moreover, higher accumulation was seen near the tumor site, which might be explained by the interaction of HA with the hyaluronate receptor (CD44). The effective administration of an anti-glioma siRNA in GL261 tumor-bearing mice resulted in tumor growth suppression and increased survival time. Moreover, toxicology testing on rats revealed no harmful effects on the trigeminal nerves or nasal mucosa; hence, it could be concluded that the HA/DP7-C could be a potential delivery system for siRNA delivery via the IN route for glioma treatment.

Nowadays, antisense nucleotides (ASOs) have gained prominence in the treatment of a variety of illnesses, including neurodegenerative and neuromuscular disorders [201]. But still, the clinical effectiveness of ASOs is limited by their fast clearance and vulnerability to nucleases [202]. In a study, using the emulsification solvent evaporation process, nasal mucoadhesive microparticles were formulated for the delivery of phosphorothioate ASO (PTO-ODNs) [209]. PTO-ODN microparticles were either coated with the mucoadhesive polymer polycarbophil–cysteine (PCP–Cys) or with unmodified PCP and reduced glutathione (GSH). They showed slower clearance from the nasal cavity, a longer contact time with the nasal mucosa, high stability, better ASO penetration, and controlled release. The nano/microparticles resulted in a 2.2-fold increased absorption from the nasal mucosa, suggesting their suitability as carriers for IN delivery of ASOs. Vetter et al. investigated the role of thiolated polycarbophil as a multifunctional adjuvant in the IN administration of ASOs [210]. They found that the ASO uptake from the nasal mucosa increased by 1.7-fold in the presence of 0.45% thiolated polycarbophil and 0.5% glutathione. These findings suggested that thiolated polycarbophil/GSH might also be a viable excipient for nasal delivery of ASOs and useful in enhancing transport across the nasal mucosa without affecting its morphology.

Neurosteroids, generated in the glial cells and neurons in the CNS, are powerful endogenous neuromodulators and have been found to have diverse functions in the CNS [211,212]. Sex hormones such as progesterone, testosterone, and oestradiol have been reported to have specialized functions in normal or pathological brain function, such as impacts on cognition, anxiety, depression, appetite management, emotion, motivation, and motor abilities [25,213,214,215,216,217,218,219,220,221,222,223]. There is substantial evidence that these steroids are absorbed into nasal mucosal capillaries and subsequently transferred from venous circulation through the BBB into the brain, but a portion of the dose is delivered straight to the brain, circumventing the BBB upon IN administration. As a result, after deposition into the nose, the relative concentrations of these steroids in particular brain areas (e.g., olfactory bulb) closer to the nasal cavity were found to be greater [213,214].

Pregnenolone, the precursor of neuronal progesterone, is acquired from the circulation or by local de novo synthesis from cholesterol and is then converted to progesterone by 3-hydroxysteroid dehydrogenase [25,213,214]. In research conducted by Ducharme et al., radio-labeled pregnenolone and progesterone administered intranasally in an oleogel formulation (a viscous castor oil mixture, MetP Pharma AG, Emmetten, Switzerland) appeared to target the brain more efficiently than IV treatment in CD-1 mice [215]. Pregnenolone-induced memory improvement and anxiety reduction associated with progesterone indicated that therapeutic amounts of neurosteroids were achieved in the brain following IN administration using these formulations. Similarly, IN administration of progesterone (0.5, 1.0, or 2.0 mg/kg) using proprietary MetP Pharma oleogel, to male Wistar rats (5 μL each) resulted in an immediate substantial rise in dopamine levels in the basolateral amygdala and a delayed significant increase in the neostriatum. Based on the findings, the authors concluded the potential of progesterone in increasing dopamine levels in the brain. Another study developed oestradiol and progesterone formulations by dissolving them in ethanol with randomly methylated β-cyclodextrin (RAMEB) (molar ratio 1:2) to form inclusion complexes to improve their solubility [217,218]. To achieve the final oestradiol and progesterone formulations, ethanol was evaporated under a moderate nitrogen stream (50 °C), and the inclusion complexes were dissolved in sterile saline. Two percent (*w*/*v*) of oestradiol and 9% (*w*/*v*) of progesterone formulations attained C_max_ levels in plasma and CSF within 15 min after IN administration in rats. Similarly, Wang et al. used the microdialysis method to investigate the absorption of oestradiol in rats using formulations based on RAMEB inclusion complexes [219]. The results showed that oestradiol was carried into CSF via olfactory neurons, indicating a direct transport pathway from the nose to CSF.

Testosterone is an endogenous steroid that has essential functions in both peripheral tissues and the CNS. IN administration of testosterone in CD-1 mice using Noseafix^®^ (patented gel formulation) resulted in brain targeting, especially in the olfactory bulb, hypothalamus, striatum, and hippocampus [220]. Silva et al. delivered testosterone intranasally to anesthetized male rats, and its effects on the activity of dopaminergic and serotonergic neurons were examined. Testosterone treatment using proprietary MetP Pharma oleogel in both nostrils of Wister rats resulted in increased levels of dopamine and serotonin in the neostriatum and nucleus accumbens. Based on these findings, the authors concluded that IN testosterone delivery is more effective in reaching the brain than the subcutaneous route and may be used to activate the central dopaminergic and serotonergic systems. In addition, Zang et al. reported that IN administration of testosterone dissolved in sesame oil enhances mobility, exploratory activity, and motor and grooming behavior in rats. In rats, intranasally delivered allopregnanolone at a concentration of 16 mg/mL in an aqueous solution containing 0.9% NaCl and 40% sulfobutylether-β-cyclodextrin protected rats against seizures without inducing behavioral adverse effects, indicating direct N2B transport with preferential transport to seizure-relevant brain regions [223]. Table 3 summarizes various biologics used for direct N2B delivery.

## 6. Nasally Administered Biologics Currently on the Market and in Clinical Trials

Despite the success of N2B delivery at preclinical and sometimes clinical levels, screening of the drug base bank reveals a limited number of successfully marketed biologics, as shown in Table 4. A few decades ago, the majority of marketed medications were hormones, marking the first milestone in biologic nasal delivery. For example, the peptide buserelin (gonadotropin-releasing hormone (GnRh) analogue) is delivered nasally to treat hormone-dependent metastatic prostate cancer. It is quickly broken down completely in the digestive tract when taken orally and has a bioavailability of 2–3% when administered intranasally—at least in the current formulation. The IN bioavailability of buserelin is also substantially lower compared to subcutaneous injection (70%), but that is still effective against advanced prostate cancer and endometriosis [268]. Desmopressin, an antidiuretic hormone, is sold under the brand names Minirin™, Ddavp™, Noctiva™, Octostim spray™, and Stimate™ for the treatment of nocturnal enuresis and central cranial diabetes insipidus. The bioavailability of desmopressin following nasal administration is 10–20 times that of oral administration [269]. Glucagon is a peptide hormone that is administered intramuscularly to treat type 1 diabetes in youth. The bioavailability of glucagon nasal powder with the absorption enhancer dodecylphosphocholine was equivalent to that of an intramuscular glucagon injection [270]. Both intramuscular and IN (Baqsimi™) formulations of glucagon result in similar pharmacokinetic profile (reaching C_max_ with 5 min). Nafarelin, commonly known as Synarel™, is a IN GnRH agonist spray that is used to treat endometriosis and early puberty [271]. It is also used to treat uterine fibroids, to control ovarian stimulation during IVF, and as part of transgender hormone therapy. IN salmon calcitonin (Miacalcin™ or Fortical™) is a peptide approved by the FDA for the treatment of osteoporosis in women over the age of 50 [272]. Table 4 lists intranasally administered biologics available on the market.

Table 5 lists the current status of clinical trials using biologics for the N2B delivery for several CNS disorders.

## 7. General Comments and Concluding Remarks

N2B delivery is a non-invasive, convenient, and patient-friendly route of drug administration that has the potential to provide a fast onset of action with accurate drug targeting and reduced systemic side effects. Moreover, it can provide the added advantage of transporting the drug into the brain directly by avoiding the BBB. However, the clinical translation of IN formulations still has some way to go. N2B delivery has numerous limitations, including mucociliary clearance, enzymatic degradation, and possible drug/formulation-related mucosal toxicity and neurotoxicity, that limit its potential uptake by the pharmaceutical industry. There are formulation-specific problems (e.g., availability/suitability of excipients, stability, scale-up, etc.), which can make it less attractive in comparison to other well-established routes. Although options to address some of these issues are already available, concerns remain. For example, the application of the nanoparticles in IN formulations requires long-term biosafety data. Although most nanoparticles for IN delivery are formulated with well-studied natural or biocompatible (PLGA, PCL, etc.) polymers or lipids, the impact of other components in the formulation is frequently ignored. For instance, the long-term safety and impact of lectins on the nasal mucosa from formulations designed for chronic therapies are still understudied, which needs to be established for these bioligands to be accepted by scientists and industry. Similarly, the evidence available on the biocompatibility and toxicity of inorganic nanoparticles in IN formulations is at best controversial, which limits their application as viable carriers in N2B drug delivery. Moreover, the fate of nanoparticles, especially in long-term therapy, needs careful attention, as their accumulation in the brain or circulation in extracellular fluids would not be desirable.

Another significant challenge in N2B drug delivery originates from the limited understanding of the spatial distribution of drugs in the brain tissue upon application. Although direct routes involving neuronal pathways (e.g., olfactory and/or trigeminal nerve systems) and indirect absorption via vasculature and lymphatic systems are commonly proposed, the exact mechanisms of drug absorption are anything but fully established. A thorough understanding of these routes and whether one or various mechanisms contribute to drug transportation simultaneously needs to be established for the successful development of IN formulations. Another challenge in establishing IN delivery as a major platform is the availability of in vivo models. The use of rodents is widespread while studying IN delivery, but anatomical differences between human and rodent noses can make pre-clinical results difficult to translate. Although progress has been made in developing fluorescent probes, imaging techniques, and in vitro/in vivo models, a concerted effort is still required to gain further understanding of these mechanisms.

Mucociliary clearance is also a major challenge in the nasal administration of applied formulations. It is difficult to resolve this via traditional formulations such as sprays. Hence, formulators need to explore novel ways that can increase the residence time of the formulation within the nasal cavity. The application of bioadhesive hydrogels or oleogels can rectify this to a certain extent. In situ gel-forming formulations can allow easy application and enhance the retention of the formulation without needing highly specialized delivery devices. However, the suitability of excipients in the IN delivery once again needs to be established to increase the available options.

Also, in numerous instances, the drug reaching the CNS can be very small compared with the amount of drug applied in the nasal cavity. The major issue with N2B delivery is the difficulty in reaching the olfactory region and then limited absorption (for peptides and protein-based drugs) once it has reached there. The application of absorption enhancers and mucoadhesive polymers can help in addressing these challenges, but their long-term toxicity in chronic nasal therapy remains unexplored. It is important to establish this before new products are made available for the N2B delivery of drugs that are not readily absorbed across the nasal mucosa. Similarly, other strategies, including charge neutralisation, solubilisation with additives, and stealth approaches to evade immune clearance, also need to be investigated specifically for their application in N2B delivery systems.

The outlook for N2B drug delivery in the pharmaceutical landscape is marked by both challenges and promising advancements. While hurdles exist, ongoing studies are anticipated to address limitations and contribute to a better understanding of the mechanisms associated with this route of drug delivery. We believe that the focus of future efforts in this area will be on elucidating the mechanisms involved in drug delivery from the nasal cavity to the brain, with an emphasis on the importance of properly designed formulations. The choice of excipients, along with their acceptance by the pharmaceutical industry and regulatory authorities, would allow formulators to work in a wider space, which would help to tune current approaches and develop novel delivery platforms.

This review discussed the challenge of delivering pharmaceutical actives, especially molecules such as peptides and proteins, to the brain while circumventing the BBB. The N2B route can be highly promising where the application of nanocarriers, targeting ligands, and mucoadhesive agents can assist drug transport through the nasal mucosa and promote delivery to the brain. The current landscape of N2B drug delivery research reveals a substantial concentration of small-molecular-weight drugs, along with some peptides and proteins, at the preclinical stage. However, the translation of these promising preclinical findings to clinical applications has been notably limited, encompassing only a few specific types of drugs. The bottleneck appears to stem from challenges associated with finding appropriate excipients, moving laboratory-level formulations to large-scale production, the availability of suitable animal models, and ensuring drug uniformity and stability in formulations. There is no doubt that the ongoing development of formulation technology and our improved understanding of the excipients will yield the development of novel strategies for N2B drug delivery that can offer potential solutions to the longstanding challenges associated with delivering high-molecular-weight drugs to the brain. Nonetheless, N2B delivery, especially for biologics, is still underexplored, even using currently available approaches. Undoubtedly, an intensified approach to expanding and clinically applying available delivery strategies to a wider array of drugs is needed, considering the various advantages that this route offers. There are a number of small-to-large molecular-weight drugs currently in clinical trials, which is encouraging, and it provides an optimistic outlook for the N2B delivery of the pharmaceutical actives. It can be anticipated that the ongoing advancements in formulation technologies and excipient availability will pave the way for a more diversified and clinically impactful N2B drug delivery approach in the near future. 

## Figures and Tables

**Table 1:** List of biologics for the treatment of CNS disorders.

Therapeutic Moiety	Applications in CNS Diseases	Ref.
***Peptides:*** *Modulate neurotransmitter function, regulate signalling pathways, prevent protein misfolding and aggregation*		
Insulin	Alzheimer’s disease	[7]
NAP neuropeptide	Alzheimer’s disease	[8]
Vasoactive intestinal peptide	Neuroprotection	[9]
Urocortin	Alzheimer’s disease	[10]
Leucine-enkephalin	CNS disorders	[11]
MS-1 (amino acid sequence CRGGKRSSC) novel peptide ligand	Multiple sclerosis	[12]
Gly14-humanin	Alzheimer’s disease	[13]
Oxytocin	Autism spectrum disorders	[14]
***Proteins:*** *Target specific receptors, enzymes, and transporters in the CNS, regulate synaptic transmission, promote cell survival and differentiation*		
Neurotrophic factors (NGF, BDNF, CNTF, NT-4)	Focal ischemia, neuronal death, traumatic brain injury	[15]
Growth factors (IGF-1, TGF-α, FGF, HNGF, VEGF, BFGF)	Alzheimer’s disease, stroke, Parkinson’s disease, epilepsy, traumatic brain injury	[15,16]
Erythropoietin	Traumatic brain injury	[15]
Ovalbumin	Neurodegenerative disorders	[17]
***Nucleic acid-based drugs:*** *Regulate gene expression, modulate RNA splicing, and translation*		
Mac-1 siRNA	CNS disorders	[18]
GFP-mRNA luciferase mRNA	CNS disorders	[19]
Plasmid DNA	Neurodegenerative disorders	[20]
499-siRNA or 233-ASO	Parkinson’s disease	[21]
anti-eGFP siRNA and dsDNA	Alzheimer’s disease	[22]
anti-ITCH siRNA	CNS disorders	[23]
siRNA or dsDNA	Neurodegenerative disorders	[24]
***Steroids:*** *Regulate inflammation, protect against oxidative stress, promote cell survival and differentiation*		
Sex hormone (progesterone, testosterone, oestradiol)	CNS disorders	[25]
Thyrotropin-releasing hormone (TRH)-peptide	Epilepsy	[26]
Melanocortin-4 receptor antagonist	Neuropathic pain	[27]
***Antibodies:*** *Target pathogenic proteins, modulate immune responses, promote cell clearance and phagocytosis*		
Antibody fragment (TNF-a inhibitory single-chain Fv antibody fragment)	Parkinson’s disease Alzheimer’s disease, MS	[28]
RNA based aptamers	CNS disorders	[29]
Full-length anti-Nogo-A antibody	Ischemic stroke	[30]

**Table 2:** Examples of various approaches and excipients used in the IN delivery of APIs for brain targeting.

Enhancer	Drug	Species/In Vitro/In Vivo	Comments	Ref.
***Surfactants***				
Laurate sucrose ester	Sumatriptan succinate	Rat	Promising IN absorption enhancer for poorly permeable drugs	[128]
Pluronic F-127	Donepezil	In vitro Ex vivo In vivo (pig)	Adequate mucoadhesive properties; improved drug permeation through nasal mucosa	[129]
Rhamnolipids (biosurfactant)	Dextran	In vitro	Safe and effective excipient for the improvement of mucosal absorption of macromolecules Concentration-dependent permeability effect; higher permeability observed for lower MW dextran	[130]
***Cell-penetrating peptides (CPP)***				
Low-molecular-weight protamine (LMWP)	Bovine serum albumin Peroxidase β-galactosidase	Mouse	Successful nose-to-brain delivery with selected enzymes retaining their biological function after delivery	[131]
Penetratin	Insulin	Rat	Efficient intranasal absorption of insulin up to deeper regions of the brain such as the hippocampus and cerebellum, reduced systemic exposure with D-penetratin	[7,132]
Polyethylene glycol–polycaprolactone copolymers conjugated with Tat peptide (MPEG–PCL–Tat)	siRNA Dextran	Rat	The CPP-modified nanomicelles improved transport along the olfactory and trigeminal nerve pathway due to their high nasal mucosa permeability	[133]
L-penetratin	Leptin	Rat	Improved nasal absorption with co-administration of L-penetratin; increased plasma concentrations and brain distribution (particularly in the olfactory bulb and hypothalamus); no toxic effect on epithelial cells	[134]
Exendin-4–CPP conjugate	Exendin-4, a glucagon-like protein-1	Mouse	Improved the scope for treatment of progressive cognitive dysfunction	[135]
***Bile salts***				
Sodium-ursodeoxycolate/ Sodium taurocholate	Zidovudine	In vitro In vivo (rat)	Results indicating antiviral drug targeting of macrophages in CSF using nano-systems coated with these bile salts	[136]
***Polymeric system***				
Chitosan nanoparticles	Bromocriptine	Mouse	Significant improvement of bromocriptine bioavailability in the brain following IN administration of drug-loaded chitosan nanoparticles	[137]
Chitosan glutamate microspheres	Rokitamycin	In vitro In vivo (rat)	Improved dissolution rate and successful nose-to-CSF delivery of the drug molecules	[138]
Chitosan nanoparticles	Venlafaxine	Ex vivo In vivo (rat)	Higher drug transport efficiency and direct brain transport percentage with these nanosystems in comparison to other formulations	[57]
Chitosan glutamate (CG)/chitosan base (CB)/hydroxypropyl methylcellulose (HPMC) microparticles	Zolmitriptan	In vitro Ex vivo In vivo (rat)	Among the investigated nasal formulations, CG-based microparticles showed the best efficacy in promoting the central uptake of zolmitriptan	[139]
Chitosan + glycerophosphate + magnesium chloride hydrogels	Exenatide	In vivo (rat)	Presence of MgCl_2_ led to improved exenatide stability, extended gelling time, improved transepithelial transport, biodistribution and bioavailability	[140]
PEG-PCL- or stearate-modified arginine-rich-CH2R4H2C peptide	Dextran	In vivo (rat)	Effective N2B delivery with less distribution to other peripheral tissues than that with IV administration; stearate-CH2R4H2C is more suitable for drug transport to the forebrain while PEG-PCL-CH2R4H2C allows for targeted transport to the hindbrain	[141]
Poly (lactic-co-glycolic acid) (PLGA) nanoparticles conjugated with glutathione	Paclitaxel	In vitro Ex vivo In vivo (rat)	Efficient brain delivery following nasal administration of drug-loaded- conjugated carrier; glutathione shows to be a suitable vector for the successful transport of poorly bioavailable drug to the brain	[142]
Alginate–chitosan nanoparticles	Venlafaxine	Ex vivo In vivo (rat)	Improved drug’s pharmacodynamics when compared to IN solution and oral tablet. Also, greater brain/blood drug ratios with nanoparticles	[143]
Polycaprolactone nanoparticles	Aripiprazole	In vitro Ex vivo In vivo (rat)	Better drug distribution in the brain than IV. Nasal toxicity study indicated the safety of the developed nanoparticle formulation	[144]
Glycol chitosan-coated nanostructured lipid carrier	Asenapine	In vitro In vivo (rat)	Promising delivery system for the brain transport via the IN route, with better pharmacokinetic and safety profile; approximately, 2.3- and 4-fold higher systemic and brain bioavailability respectively for the drug-loaded carrier.	[145]
Chitosan-coated solid lipid nanoparticles	BACE1 siRNA + RVG-9R (cell-penetrating peptide) complex	In vitro	Mucoadhesive properties and prolonged residence time in the nasal cavity; improved siRNA epithelial cell (Caco-2) permeation after release from coated particles	[146]
PLGA nanoparticles embedded in in situ poloxamer 407^®^ (P407) gel	Rivastigmine hydrogen tartrate (RHT)	In vitro Ex vivo	Nanocomposites showed higher amounts of drug permeation through sheep nasal mucosa than plain drug gel	[147]
Chitosan nanoemulsions	Kaempferol	In vitro Ex vivo In vivo (rat)	Higher permeation, brain bioavailability, and efficacy of the drug when compared to free drug or non-mucoadhesive nanoemulsions; histopathological examination showed safety of nanoemulsion for nasal mucosal and ability to preserve drug antioxidant capability	[148]
Polycarbonate nanoparticles	Apomorphine	In-vitro In-vivo (Rat)	Improved brain bioavailability	[149]
Lectin-modified PEG–PLGA nanoparticles	Basic fibroblast growth factor	In vitro In vivo (rat)	Enhanced spatial memory, bioavailability, therapeutic activity, and reduced side effects	[150]
N-trimethyl chitosan nanoparticles	Leucine-enkephalin	In vitro Ex vivo In vivo (rat)	Improved brain uptake, antinociceptive effect and therapeutic activity	[151]
Chitosan based nanoemulsion gel	Naringenin	In vitro Ex vivo In vivo (rat)	Increased brain bioavailability and showed no toxicological or inflammatory response	[152]
Gelatin nanoparticles	Osteopontin	Rat	Gelatin microspheres enhanced the neuroprotective effects of osteopontin	[153]
***Lipid-based systems***				
Chitosan-coated nanostructured lipid carrier.	hIGF-I	In vitro	Enhanced biodistribution and facilitated efficient drug delivery	[154]
	Glial cell-derived neurotrophic factor (hGDNF)	Ex vivo In vivo (rat) In vivo (rat)	Improved behavioral patterns and neuroprotection	[155]
Gelatin-NLCs	Basic fibroblast growth factor	In vitro (mouse)	Improved brain bioavailability, target efficiency and therapeutic effects	[156]
Oil-in-water nanoemulsion	Cyclosporine-A	In vivo (rat)	Improved targeted drug delivery and bioavailability	[157]
Chitosan NLCs	Glial cell-derived neurotrophic factor	Rat	Enhanced brain distribution in the PD model	[126]
Cationic liposomes	GFP-mRNA luciferase mRNA	Rat	Higher expression of GFP-mRNA expression post 24 h compared to naked mRNA	[21]
***Miscellaneous***				
Capmul MCM (oil) + labrasol (surfactant) + transcutol P (co-surfactant) + Carbopol 934P (mucoadhesive agent) + Pluronic F127, F68 (gelling excipient)	Nimodipine	Rat	A combination of Pluronics and Carbopol 934P can significantly increase the N2B delivery of nimodipine	[114]
Human serum albumin + chitosan	Sulforhodamine B sodium salt	Ex vivo	Confirmation of mucoadhesive properties of chitosan; added advantage of opening of tight junctions	[158]
Cationic liposomes	Ovalbumin	Rat	Brain delivery of model protein via the nasal olfactory route and extended brain residence time of the delivered biomolecule	[121]
Chitosan + hydroxypropyl-b-cyclodextrin microemulsions	Buspirone hydrochloride	In vivo (rat) Ex vivo	Direct N2B transport of 88% buspirone following IN administration	[159]
*Delonix regia* gum-coated nanostructured lipid carriers (DRG-NLCs) [NLCs comprising of glycerol monostearate (solid lipid); Capryol 90 (liquid lipid); soya lecithin (surfactant); poloxamer 188 (cosurfactant)]	Ondansetron	In vitro Ex vivo In vivo (rat)	Rapid drug transport and improved bioavailability to the brain by IN administration of DRG-NLCs	[160]
Liposomes (cholesterol + egg phosphatidylcholine)	Quetiapine	In vitro Ex vivo In vivo (mouse)	Better potential for quetiapine N2B delivery with formulated liposomes in comparison to simple drug dispersions	[161]
Flexible liposomes (soya phosphatidylcholine + cholesterol + propylene glycol + water)	Galanthamine hydrobromide	In vivo (rat)	Improved efficiency of drug activity in the brain after IN administration in comparison to oral. Increased C_max_ and AUC_0→10_, and reduced drug cell cytotoxicity with nasal delivery using liposome carrier	[162]
Ion-activated deacetylated gellan gum (DGG) in situ gel incorporating resveratrol nanosuspensions	Resveratrol	In vitro In vivo (mouse)	Direct transport of drug (78%) to the brain via the nasal cavity	[163]
Flaxseed oil containing cationic DOTAP nanoemulsions	Anti-TNFa siRNA	In vitro In vivo (rat)	Enhanced cell (J774A.1 murine macrophage) uptake by endocytosis of siRNA in comparison to Lipofectamine^®^ formulations; higher relative gene silencing effect in lipopolysaccharide (LPS)—stimulated macrophages	[164]
Agomelatine-nanoemulsion in situ poloxamer-407 gel (Ag-NE-gel) + 0.5% chitosan	Agomelatine	In vitro Ex vivo In vivo (Rat)	Improved drug bioavailability in the brain following IN administration; rapid gel erosion, faster drug release from NE and better drug permeation through the olfactory epithelial layer	[165]
Nanoliposomes (phospholipon 90G + cholesterol + Tween 80)	Lamotrigine	In vitro Ex vivo	High drug release; enhanced drug permeation across the nasal mucosa	[166]
PGLA Nanoparticles		In vivo (rat) In vitro	Increased bioavailability and permeation in the brain	[115]
Poly (lactic-co-glycolic acid) nanoparticles (NPs)	Diazepam	In vivo (sheep) In vivo (rat)	Potential carrier for N2B delivery of outpatient management of status epilepticus	[116]
Thiolated chitosan nanoparticles	Galantamine	In vivo (mouse)	Significantly improved efficacy (*p* < 0.05) compared to oral administration.	[117]
Polycaprolactone nanoparticles	Melatonin	In vitro In vivo (rat)	Increased apparent solubility (~35 fold), effective treatment for glioblastoma	[118]
Nanoemulsion [triacetin (oil phase) + Tween 80 (surfactant) + PEG-400 (co-surfactant)]	Letrozole	In vitro ex vivo In vivo (mouse)	Enhanced release compared to drug suspension. Higher bioavailability in the brain and improved anticonvulsant drug effect with the IN nanoemulsion in comparison to intraperitoneal route	[167]
Pluronic F-127 + Carbopol 974P thermoreversible gel	Levetiracetam	In vitro In vivo (mouse)	Higher cerebral concentrations following IN administration and, similar plasma PK profile to IV. No change in cell viability in nasal and lung cells in the presence of drug–gel formulation	[168]
Pluronic F-127 + Carbopol 934 thermoreversible gel	Naratriptan	In vitro Ex vivo	Carbopol acts as both a mucoadhesive agent and a penetration enhancer	[169]
Microemulsion of Capmul MCM EP (oil) + surfactant mix (labrasol + Tween 80 + transcutol-P) + DW or mucoadhesive ME with chitosan or methyl-b-cyclodextrin	Quetiapine	Ex vivo In vivo (rat)	Superiority of chitosan ME formulation; enhanced brain uptake following IN administration	[170]

**Table 3:** Intranasal delivery of biologics for CNS delivery.

Active	Mol wt. (kDa)	Carrier System	Formulation Type	Species	Observations	Potential Treatment/Application	Ref
Arginine-vasopressin (AVP)	1.1	--	AVP (10 IU) in 10 µL sterile water was administered via Rhintile (Ferring, Germany) (0.2 mL per nostril)	Human	Amplified P3 component of event-related potentials (ERPs) in brain	Increased brain activity	[224]
Angiotensin II (ANG II)	1.0	ANG II diluted in sterile 0.9% NaCl solution	Spray-single intranasal puffs of 100 μL (dose of 400 μg ANGII) within 1 min	Human	Acutely increased blood pressure by directly influencing the CNS; maintained plasma norepinephrine levels	Regulation in blood pressure in CNS	[225]
Activin A and Serpin B2	26.2 45–47	Tetradecyl-β-D-maltoside (TDM)	1 μg each of Activin A, SerpinB2, GFP, ΔNpas4, or 1 μg Activin A + 1 μg SerpinB2 in 20 μL, with or without TDM	Mouse	Maintains the structural and functional integrity of neurons, slows down the progressive cognitive disease	Alzheimer’s disease, Huntington’s disease, and amyotrophic lateral sclerosis, Parkinson’s disease, brain damage and stroke	[226]
Apomorphine	0.285	Polycarbonate nanoparticles	Free AMP/polymer conjugated AMP in PBS	Mouse	Improved bioavailability	Parkinson’s disease	[149]
Anti-trail monoclonal antibody	40	PLGA nanoparticles and NLCs	Solution	Mouse	Reduced neuroinflammation	Alzheimer’s disease, Parkinson’s disease, Epilepsy	[197]
Brain-derived neurotrophic factor (BDNF) with co-delivery of simvastatin	14	PEG-PLA polymersomes and pluronic F127	Polymersome formulations: prepared at 1 μg/mL BDNF loading, simvastatin concentration varied at 5, 10, and 20 μg/mL	Mouse	Maintains and protects neurons. Attenuates lipopolysaccharide-induced inflammation	Alzheimer’s disease, Parkinson’s disease, Huntington’s disease, Multiple sclerosis	[227]
BDNF	26.9	---	70 μg of neurotrophic factor in 70 μL sterile PBS	Rat	Neuroprotective improvement	Parkinson’s disease, Multiple sclerosis	[15]
Basic fibroblast growth factor (BFGF)	18	Gelatin NLCs	Suspension (2 mg/mL)	Rat	Neuroprotective improvement	Parkinson’s disease	[156]
Calcitonin gene-related peptide	3.8	--	1 μg CGRP in 50 μL water, 10 injections (5 μL each), alternating nostrils, 2 min interval	Rat	Reduced vasospasm, improved cerebral blood flow, reduced cell death, and stimulated angiogenesis following subarachnoid haemorrhage	Cerebral vasospasm	[228]
		Acidic and basic gelatin	Salmon calcitonin gelatin microspheres (swelling in pH 7 PBS) and sCT solution (0.1 mL/kg, pH 7.0 PBS) via intranasal route at 15.0 U/kg	Rat	Improved nasal absorption of the drug	Improved nasal absorption of the drug	[229]
		Pheroid™	Pheroid vesicles and microsponges loaded with salmon calcitonin, *N*-trimethyl chitosan chloride (TMC) saline solution	Rat	Improved nasal absorption of the drug	---	[230]
Cholecystokinin-8	1.1	---	CCK8 dissolved in sterile water and dose of 5 µg was sprayed in each nostril (solution)	Human	Amplified P3 component of auditory evoked potentials as well as plasma corticotropin levels.	Increased brain activity	[231]
Cystatin C-peptide	13	---	20 µg of CysC-AβBP and scrambled peptide (solution)	Mouse	Reduces amyloid aggregates and improves memory	Alzheimer’s disease	[232]
Caspase-1 inhibitor	17	--	Caspase-1 inhibitor (5 µg/µL) administered via nose drops (2.5 µL/drop) over 20 min, for a total volume of 20 µL (solution)	Rat	Decreases hippocampal neuronal loss and improves neurocognitive action	Global cerebral ischemia	[233]
TNF-α siRNA	NA	Cationic nanoemulsion	5 μL/nostril of siRNA nanoemulsion or saline solution, 1 min hold between doses	Rat	Site-specific downregulation of TNF-α cytokines	Prevention of neuroinflammation	[164]
Erythropoietin (EPO)	30.4	---	20 µL volumes of 0.6, 2.4, 6, and 12 U rhEPO/20 µL sterile saline delivered to each nostril	Mouse	Improves neurological- function, memory alterations, reduces infarct volume and improves neurologic function	Alzheimer’s disease, cerebral ischemia, epilepsy	[234]
			EPO diluted in PBS (pH 7.0) at 0.15 Mm. Administration at 125 and 250 µg/kg	Mouse	Alleviated memory alterations, oxidative stress, neuroinflammation, apoptosis induction, and amyloid load	Cerebral ischemia, neuroinflammation, Alzheimer’s disease	[235]
Exendin (9–39)	4.186	Pegylated Ex-4	PEGEx-4 analogs in 100 mM PBS pH 7.4	Rat	Reduces the insulin responses to enteral glucose	Congenital hyperinsulinism	[236]
Radio-labeled exendin (9–39) (I-Ex)	3.4	Cyclodextrin	2 μL of phosphate buffer/normal saline with 500,000 cpm of I-Ex ± 5% β-cyclodextrin (CD)	Mouse	Improved brain uptake	CNS disorders	[187]
Full-length IgG	150	---	Aβ25–35 + Glu-Ab: 300 μg/kg Glu-Ab water solution Aβ25–35 + γ-globulin: 300 μg/kg rabbit γ-globulin water solution	Rat	Anti-amnesic effect in an AD model; improved conditioned passive avoidance response following ischemia	Alzheimer’s disease, ischemic injury	[237]
Galanin-like peptide (radioactively labeled)	6.5	α-cyclodextrin, dimethyl β-cyclodextrin	2 μL of 250,000 cpm/μL I-GALP alone, with cyclodextrins (β-CD or α-CD), or with 1 μg/mouse unlabeled GALP solution	Mouse	Increased brain uptake	Eating regulation	[238]
H102 peptide (novel β-sheet breaker peptide)	NA	Liposomes egg phosphatidylcholine, DSPE–PEG, and cholesterol	H102 solution (2 mg/kg) with 1% chitosan, 0.1% BSA, and H102 liposomes (2 mg/kg)	Mouse	Improved spatial memory impairment and enhanced brain bioavailability	Alzheimer’s disease	[239]
Human nerve growth factor		26.5	1 M phosphate-buffered saline (PBS, pH 7.4)	Rat	Improved memory and enhanced neurogenesis	Alzheimer’s disease	[240]
Gly14-humanin (S14G-HN)	NA	Odorranalectin cubosomes	1.0 mg/kg of cubosomes (10 μL per nostril) in 10 mmol PBS (pH 7.4)	Rat	Improved brain bioavailability and therapeutic activity	Alzheimer’s disease	[241]
Insulin	5.8	Penetratin CPP	Exendin-4 solution (1 mg/mL) with L-penetratin (2 mM) ± insulin (8 IU/mL)	Mouse	Protection against neurodegeneration and improved brain bioavailability, partial neuroprotection	Alzheimer’s disease, mild cognitive impairment	[135]
		Penetratin CPP	L- or D-penetratin in pH 6.0 PBS with 0.001% methylcellulose, mixed with insulin to achieve 30 IU/mL and 2.0 mM solution	Mouse	Improved absorption of Insulin via nasal cavity	Alzheimer’s disease, mild cognitive impairment	[177]
		Liposome suspension	Suspension	Mouse	Enhanced permeation vs. insulin solution	-----	[180]
		Maltodextrin DE 8/22/ + Carbopol^®^ 974P (90/10) DE 38 + (80/20)	Powder	Rabbit	Improved brain bioavailability	Alzheimer’s disease	[242]
		Starch + sodium glycodeoxycholate (GDC) 0.08 mg/kg starch microspheres + LPC 0.05 mg/kg	Powder	Sheep	Improved brain bioavailability	Alzheimer’s disease	[243]
		Maize starch + Carbopol^®^ 974P (90/10), drum dried waxy maize starch. (DDWM)/Carbopol^®^ 974P (90/10) or a spray-dried mixture of Amioca starch/Carbopol^®^ 974P (25/75)	Powder	Rabbit	Improved brain bioavailability	------	[183]
		Poly (N-vinyl pyrrolidone)-based nanogels	Gels	Mouse	High biocompatibility, no immunogenicity, rapid clearance within 24 h, enhanced delivery to all brain regions vs. free insulin	Alzheimer’s disease	[179]
		Polyacrylic acid	Gels	Mouse	Improved absorption of Insulin via nasal cavity	------	[181]
Insulin-like growth factor I	7.650	--	10 mM sodium succinate buffer containing 140 mM sodium chloride at pH 6.0	Mouse	Enhances neurological function and prevents apoptosis, reduced infarct volume/brain oedema and enhanced neurologic function; in neonatal rats; prevented apoptosis after hypoxic-ischemic damage	Focal cerebral ischemic damage	[244]
			PBS solution containing 0.25% BSA	Mouse	Reduction in stroke volume and improved behavioral patterns	Alzheimer’s disease, stroke	[188]
			PBS solution containing 0.25% BSA	Rat	Improved neurobehavioral performance, inhibition of apoptotic cell death	Cerebral hypoxia-ischemia	[189]
Interferon β1b	18.5	--	Aqueous solution of human recombinant IFN-β and [^125^I]-labeled human recombinant IFN-β at pH 4.	Adult cynomolgus monkey	Showed central distribution of the macromolecule along the olfactory and trigeminal pathway	Multiple sclerosis	[245]
	19.86		^125^I-IFNh-1b + rhIFNh-1b solution (1.53 mg/mL)	Rat	Produced tyrosine phosphorylation of IFN receptor in the CNS	Multiple sclerosis	[246]
Interleukin-1 receptor antagonist	17	N-trimethyl chitosan nanoparticles	Leu-Enk-loaded TMC nanoparticles (0.1 mg/mL in PBS pH 6.8) via a 50 μL Hamilton micro syringe	Rat	Protects neurons and improves neurological deficit	Cerebral ischemia	[151]
Leptin	16	Sodium taurodihydrofusidiate	50 μL of 0.2, 0.1, and 0.03 mg/kg leptin solutions in 0.9% NaCl with 1% STDHF	Rat	Inhibits appetite	Obesity	[247]
mi R124	7.20	PEG-PLGA nanoparticles	Emulsion	Rat	Relieved symptoms of cerebral ischemia-reperfusion damage, provides neuroprotection	Neurodegenerative diseases	[206]
miR132	14.084	Fatty acid-modified octa-arginine CPP nanocomplexes	Suspension	Rat	Improved learning and memory	Alzheimer’s disease	[207]
m-RNA		Cationic liposomes	Solution	Rat	Improved brain bioavailability	Alzheimer’s disease	[20]
NAP neuropeptide	0.825	Lactoferrin-conjugated PEG–PCLA	20 μL nanoparticle solution containing 5 μg coumarin-6	Rat	Improves neuroprotection and memory	Alzheimer’s disease, schizophrenia, frontotemporal dementia Huntington’s disease	[248]
Neuropeptide Substance P	1.347	Gelatine-cored nanostructured lipid carriers	Suspension	Rat	Demonstrated behavioral improvement and initiation of dopaminergic neuron recovery	Parkinson’s disease	[249]
Neurotrophin-4	22.4	--	70 μg of neurotrophic factor in 70 μL sterile PBS	Rat	Improves neuronal survival	Multiple sclerosis	[15]
Neurotoxin I	6.9	PLA nanoparticles	NT-I-NPs (45 mg lyophilized nanoparticles dissolved in 0.15 mL PBS with 1:1 polysorbate 80	Rat	Enhanced brain bioavailability and facilitated brain transport of NT-I	Centrally active peptides	[250]
Neurotoxin 1	6.95	PLA nanoparticles coated with polysorbate 80	NT-P-NP (45 mg lyophilized nanoparticles in 0.15 mL PBS with 1:1 polysorbate 80, 17 mg NT/kg) and free NT solution	Mouse	Inhibits neurotransmission	Pain management	[251]
NR2B9c	0.977	WGA-functionalized PLA–PEG NPs	NR2B9c-NPs and WGA-NPs dispersed in physiological saline	Rat	Protects neurons against excitotoxicity, decreases ischemic brain injury and offers neurological function deficits	Ischemic stroke	[252]
Oxytocin	1	Oxytocin	Spray	Monkey, rabbit	Reduces anxiolytic effects social stress and enhances empathy. Increased trust; decreased stress-related cortisol; enhanced capacity to predict others’ mental states; slowed amygdala response to fear in GAD; improved emotional identification in autism	Autism spectrum disorder, schizophrenia	[253]
			OXT dissolved at 40 mg/mL in purified water	Rat	Improved brain bioavailability	Autism	[254]
			Intranasal spray or nebulizer	Monkey	Influences social cognition and behavior, increased level of oxytocin in CSF	Autism spectrum disorder, schizophrenia	[255]
Orexin-A (hypocretin-1)	3.5	--	Solution in PBS (spray)	Monkey	Reverses sleep deprivation	Narcolepsy	[256]
		1% and 5% phenylephrine	Mixture of unlabeled and ^125^I-labeled neuropeptide dissolved in PBS (solution)	Rat	Enhanced CNS targeting	Autoimmune disorders, Alzheimer’s disease or meningitis	[257]
Ovalbumin	45	Cationic liposomes	Solution in PBS	Rat	Improves bioavailability	Immunity booster	[121,258]
Pituitary adenylate cyclase-activating peptide (PACAP)	4.5	---	Aqueous solution with NaCl, citric acid monohydrate, disodium phosphate dehydrate, and benzalkonium chloride	Mouse	Enhances cognitive function Stimulated non-amyloidogenic processing and improved cognitive function.	Alzheimer’s disease, cerebral ischemia	[259]
Phosphorothioate antisense oligonucleotides (PTO-ODNs)	5	Polycarbophilcysteine or unmodified PCP and reduced glutathione (GSH)	Emulsion	Porcine nasal mucosa (in vitro/in vivo)	Induced controlled release	CNS diseases	[202]
		Thiolated polycarbophil and 0.5% glutathione	Solution	Human nasal epithelial cells, Porcine nasal mucosa	Enhanced controlled release, improved uptake	CNS diseases	[209]
Ribonucleic acid (tRNA)	0.284	Cell-permeating peptide nanocomplexes	Buffered solution	Mouse	Increases potentially therapeutic miRNA	Alzheimer’s disease	[207]
[Ser(2)]exendin (1–9) +GLP 1	NA	10% β-cyclodextrin	Buffered solution	Mouse	Improved learning and memory, provides neuroprotection, lowers rates of kainic-induced apoptosis	Neurodegenerative and cognitive disorders	[186]
siRNA	13–14	MPEG-PCL-TAT nanomicelles	Alexa dextran solution	Mouse	MPEG-PCL TAT accelerated transport along the olfactory and trigeminal nerve pathways	Alzheimer’s disease, Parkinson’s disease and brain tumor	[199]
	3.5–1350	HA/DP7-C nanomicelles	Buffered solution	Rat	Inhibited tumor growth, reduced cytotoxicity	Glioblastoma therapy	[200]
		Biodegradable PAMAM dendrimer	PBS solution	Rat	Target gene knockdown and neuroprotection, reduced infarct volumes and alleviated neurological and behavioral deficits	Postischemic brain disorders	[201]
BACE1 siRNA + Rapamycin	NA	PEGylated dendrigraft poly-l-lysines	Buffered solution	Rat	Improved cognition, promoted autophagy and improved nasal adsorption	Alzheimer’s disease	[260]
i-NOS siRNA	NA	Gelatin NPs	PBS solution	Rat	Higher therapeutic potency compared to naked siRNA	Postischemic brain disorders	[261]
Thyrotropin-releasing hormone	0.362	D, L PLA NPs	Suspension	Rat	Recovers neuron and improves behavior	Epilepsy, seizures	[262]
Thyrotrophin-releasing hormone analogs	NA	PLA-co-glycolide NPs	Suspension	Rat	Improved target efficiency and reduced seizures	Epilepsy, seizures	[263]
TNF-α inhibitory single-chain antibody fragment	26.3	CPP (Pz-peptide) + penetration enhancer	Buffered solution	Mouse	Enhanced cognitive performance, reduced cerebral amyloid angiopathy and amyloid plaque pathology	Alzheimer’s disease, Parkinson’s disease, multiple sclerosis	[196]
Urocortin	<10	Odorranalectin-conjugated PEG-PLGA NPs	OL-NPs dispersed in PBS Solution pH 7.4	Rat	Improved bioavailability and therapeutic action	Parkinson’s disease	[264]
Vascular endothelial growth factor	38.2	PEG-PLA NPs VEGF was radio-labeled with sodium ^125^I using chloramines T method	100 µL of VEGF solution	Rat	Develops behavior and enhances angiogenesis, decreased systemic side effects, increased neurotrophic and neuroprotective activity	Alzheimer’s disease, CNS diseases	[265,266]
Vasoactive intestinal peptide	16.6	WGA-functionalized PEG-PLA NPs	0.01 M HEPES buffer (pH 8.5) containing 0.1 mM CaCl_2_	Rat	Improved brain bioavailability and drug uptake	Alzheimer’s disease	[267]
Plasmid DNA	1950 ± 70	Poloxamer 188 and 107 with polyethylene oxide and polycarbophil	100 μL of plasmid DNA in 20 mL solution	Rat	Expression of encoded protein in brain and improved nasal adsorption	Alzheimer’s disease	[208]
Progesterone	0.314	Viscous castor oil-based gels	Oleogel	Rat	Enhanced brain bioavailability, reduced anxiety and depression	Cognitive impairment, Alzheimer’s disease, Parkinson’s disease, and brain damage	[219,220,221]
Testosterone	0.288						
Oestradiol	0.272						

**Table 4:** Intranasally administered biologics available in the market.

Drug	Drug Bank Accession No.	Biological Entity/Type	Condition/s	Brand Name	Marketed by	Market Approval Year	Ref.
Buserelin	DB06719	Protein based	Prostate cancer, breast cancer, endometriosis, Uterine fibroids	Suprefact Intranasal solution 1 mg/mL (spray)	Hoechst Canada Inc. (Quebec, QC, Canada)	1988	[273]
				Superfact Liq 1mg/mL (liquid)	Hoechst Roussel Canada Inc. (Montreal, QC, Canada)	1993	[172]
				Superfact (solution)	Sanofi Aventis	1998	[274]
				Suprecur (0.15 mg/spray)	Sanofi Aventis	2001	[273]
Desmopressin	DB00035	Peptide drug	Nocturia, central cranial diabetes insipidus	Ddvap (0.01 mg/spray)	Ferring Pharmaceuticals Inc. (Saint-Prex, Switzerland)(discontinued)	1978	[274]
				Ddvap (solution)	Sanofi Aventis	1978	[274]
				Minirin (0.015 mg/spray)	Ferring Pharmaceuticals Inc. (discontinued)	2000	[274]
				Noctiva (spray)	Avadel Speciality Pharmaceuticals LLC (Dublin, Ireland) (discontinued)	2017	[172]
				Octostim Spray (1.5 mg/mL, spray)	Ferring Pharmaceuticals Inc.	1998	[273]
				Stimate (1.5 mg/spray)	Ferring Pharmaceuticals Inc. (discontinued)	2011	[274]
Glucagon	DB00040	Protein based	Severe hypoglycemia	Baqsimi (3 mg powder)	Eli Lilly & Co. Ltd. (Basingstoke, UK)	2019	[274]
Nafarelin	DB00666	Synthetic agonist of gonadotrophin-releasing hormone	Central precocious, puberty, Endometriosis	Synarel (0.2 mg/ spray)	Pfizer Canada Ulc (Montreal, QC, Canada)	1996	[273,274]
				Synarel (spray)	G.D. Searle LLC (Skokie, IL, USA)	1990	
				Synarel (liquid)	Syntex Inc. (Houston, TX, USA)	1991	
Salmon calcitonin	DB00017	Hormone	Paget’s disease, osteoporosis	Fortical (200 IU/spray)	Upsher-Smith Laboratories (discontinued)	2005	[172,273,274]
				Fortical (200 IU/spray)	Physicians Total Care, Inc. (Tulsa, OK, USA)	2005	
				Miacalcin (200 IU/spray)	Novartis	1995	
Oxytocin	DB00107	Peptide drug	Labour induction	Syntocinon (40 IU/mL, solution, spray)	Novartis	1960	[172,273]

**Table 5:** Intranasally administered biologics under clinical investigation as enrolled at www.clinicaltrials.gov (accessed on 3 November 2023) [275].

Drug	Study Title	Disease	Status	Year	Identifier
Insulin (Humulin R^®^ U-100)	Study of nasal insulin to fight forgetfulness	Alzheimer’s disease	Phase II and III completed	December 2018	NCT01767909
Insulin	Intranasal Insulin and Post-Stroke Cognition: A Pilot Study	Stroke	Phase II completed	March 2020	NCT02810392
Insulin analogue	Efficacy and Safety of Human Insulin Versus Analogue Insulin in Hospitalized Acute Stroke Patients with Hyperglycaemia	Stroke, hyperglycemia	Phase IV completed	June 2020	NCT04834362
Insulin aspart	Intranasal Insulin and Memory in Early AD	Alzheimer’s disease	Phase I and II completed	May 2012	NCT00581867
Insulin aspart	Nasal Insulin to Fight Forgetfulness—Short-Acting Insulin	Alzheimer’s disease Mild cognitive impairment	Phase II completed	April 2019	NCT02462161
Insulin (humulin R U-100)	SNIFF 120: Study of Nasal Insulin to Fight Forgetfulness (120 Days)	Alzheimer’s disease Mild cognitive impairment	Phase II completed	December 2011	NCT00438568
Insulin (humulin R U-100) Empagliflozin	Nasal Insulin to Fight Forgetfulness—Combination of Intranasal Insulin and Empagliflozin Trial	Mild cognitive impairment Cognitive impairment Alzheimer’s disease	Phase II recruiting	Started October 2021	NCT05081219
Insulin	Treatment of Parkinson’s Disease and Multiple System Atrophy Using Intranasal insulin	Parkinson’s disease	Phase II completed	September 2015	NCT02064166
Insulin detemir	Study of Nasal Insulin to Fight Forgetfulness—Long-acting Insulin Detemir—120 Days (SL120)	Alzheimer’s disease	Phase II completed	March 2015	NCT01595646
Insulin detemir	To evaluate its defect in diseased patients	Alzheimer’s disease	Phase II completed	December 2012	NCT01547169
Oxytocin (syntocinon)	Intranasal Oxytocin for the Treatment of children and Adolescents with ASD	Autism spectrum disorder (ASD)	Phase II Completed	March 2016	NCT01908205
Oxytocin	Oxytocin and Social Cognition in Frontotemporal Dementia	Dementia	Completed	November 2010	NCT01002300
Oxytocin	To study the effect of drugs on PDD	Depression Premenstrual dysphoric disorder (PDD)	Completed	July 2016	NCT02508103
Oxytocin (syntocinon)	To evaluate the safety and efficacy of exogenous oxytocin on social cognition and behavior in patients with recent-onset schizophrenia	Schizophrenia Psychotic disorders	Early phase I completed	August 2019	NCT02567032
Oxytocin	Target Engagement and Response to Oxytocin	Schizophrenia	Phase IV active, not recruiting	Started January 2018	NCT03245437
Oxytocin and vasopressin	Effects of IN oxytocin and vasopressin on social behavior and decision making	Social behavior	Completed	December 2022	NCT04890470
Vasopressin	Effect of intranasal vasopressin on cooperative behavior of patients	Schizophrenia	Completed	November 2018	NCT04190004
Vasopressin	Intranasal Vasopressin Treatment in Children With Autism	Autism Autism spectrum disorder	Phase II and III Recruiting	February 2018	NCT03204786
Calcitonin	Long-term Safety Study of BHV-3500 (Zavegepant *) for the Acute Treatment of Migraine (* BHV-3500, formerly "vazegepant”)	Acute treatment of migraine	Phase II and III completed	December 2021	NCT04408794
	A Study to Learn About Zavegepant as the Acute Treatment of Migraine in Asian Adults	Acute treatment of migraine	Not yet recruiting	Estimated November 2023	NCT05989048
Insulin + semaglutide	Combination of Intranasal Insulin with Oral Semaglutide to Improve Cognition and Cerebral Blood Flow: A Feasibility Study	Metabolic syndrome and mild cognitive impairment (MCI)	Phase II Recruiting	Estimated start December 2023.	NCT06072963
Human fibroblast growth factor	Intranasal Human FGF-1 for Subjects with Parkinson’s Disease	Parkinson’s disease	Phase I, not yet recruiting	September 2022	NCT05493462
Foralumab	Phase 1b Multiple Ascending Dose Study of Foralumab in Primary and Secondary Progressive MS patients	Multiple sclerosis (MS)	Phase 1 (withdrawn)	Started October 2021	NCT05029609
Progesterone	To study the safety and efficacy of progesterone for the treatment of Acute Haemorrhagic Stroke	Brain injury, stroke	Phase IV (unknown status)	Started February 2020	NCT04143880

## Data Availability

Not applicable.
